# Correlation analysis of Th1/Th2 cytokines and liver fibrosis indicators in chronic hepatitis B patients

**DOI:** 10.5937/jomb0-60938

**Published:** 2026-04-15

**Authors:** Chun Yu, Meiling Chen, Xiaonan Han, Yuhang Feng, Hongwei Sun, Hong Yang

**Affiliations:** 1 The Quzhou Affiliated Hospital of Wenzhou Medical University, Department of Gastrointestinal Surgery, Quzhou City, China; 2 Western Theatre Command Air Force Hospital, Department of Laboratory Medicine, Chengdu City, China; 3 The Second Affiliated Hospital of Zhejiang University School of Medicine, Department of Gastroenterology, Hangzhou City, China; 4 The First Affiliated Hospital of Wenzhou Medical University, Department of Hepatobiliary Surgery, Wenzhou City, China; 5 The Quzhou Affiliated Hospital of Wenzhou Medical University, Department of Infectious Diseases, Quzhou City, China

**Keywords:** chronic hepatitis b, autoimmune antibodies, liver fibrosis, Th1/Th2 cytokines, hronični hepatitis B, autoimuna antitela, fibroza jetre, Th1/Th2 citokini

## Abstract

**Background:**

To investigate the potential therapeutic benefits of autoimmune antibody detection in individuals suffering from chronic hepatitis B.

**Methods:**

In the observation group, 102 patients with chronic hepatitis B who were admitted between March 2022 and March 2025 were included. Additionally, the control group consisted of 102 healthy people who were examined throughout the same time period. The two groups' autoimmune antibodies were identified, and patients in the observation group with either positive or negative autoimmune antibodies were compared in terms of liver function, liver fibrosis markers, and cytokine levels.

**Results:**

The total positive rate of autoimmune antibodies in the observation group was 26.47%, whereas it was 3.92% in the control group (P&lt;0.05). Aspartate aminotransferase (AST) and alanine aminotransferase (ALT) levels were noticeably greater in patients in the observation group with positive autoimmune antibodies than in those with negative antibodies. However, the albumin (ALB) and total protein (TP) levels were much lower in these patients than in patients with negative antibodies. A statistically significant difference was observed (P&lt; 0.05). The levels of type III procollagen (PCIII), hyaluronidase (HA), and laminin (LN) were significantly (P&lt;0.05) greater in individuals with positive autoimmune antibodies than in those with negative antibodies. Patients with positive autoimmune antibodies had considerably higher levels of interleukin-4 (IL-4), interleukin-10 (IL-10), and interleukin-6 (IL-6) than patients with negative antibodies. Patients who were negative for antibodies had significantly higher levels of interferon-y (IFN-y) (P&lt;0.05).

**Conclusions:**

Autoimmune antibodies, which influence liver fibrosis indicators and related cytokines, are present in patients with chronic hepatitis B. The identification of autoimmune antibodies in chronic hepatitis B patients can serve as a guide for evaluating illness and determining patient prognosis.

## Abstract (sr)

**Uvod:**

Cilj je bio da se ispitaju potencijalne terapijske koristi otkrivanja autoimunih antitela kod osoba obolelih od hroničnog hepatitisa B.

**Metode:**

U posmatranu grupu je bilo uključeno 102 pacijenta sa hroničnim hepatitisom B, hospitalizovanih u periodu od marta 2022. do marta 2025. godine. Kontrolnu grupu su činila 102 zdrava ispitanika pregledana u istom vremenskom periodu. U obe grupe su određena autoimuna antitela, a pacijenti iz posmatrane grupe sa pozitivnim i negativnim autoimunim antitelima su upoređeni u pogledu funkcije jetre, markera fibroze jetre i nivoa citokina.

**Rezultati:**

Ukupna stopa pozitivnosti autoimunih antitela u posmatranoj grupi je iznosila 26,47% , dok je u kontrolnoj grupi bila 3,92% (P&lt; 0,05). Nivoi aspartat-aminotransferaze (AST) i alanin-aminotransferaze (ALT) su bili značajno viši kod pacijenata sa pozitivnim autoimunim antitelima u odnosu na one sa negativnim. Suprotno tome, vrednosti albumina (ALB) i ukupnih proteina (TP) su bile značajno niže kod pacijenata sa pozitivnim antitelima, pri čemu je uočena statistički značajna razlika (P&lt; 0,05). Nivoi prokola tipa III (PCIII), hijaluronske kiseline (HA) i laminina (LN) su bili značajno (P&lt;0.05) viši kod pacijenata sa pozitivnim autoimunim antitelima u poređenju sa onima sa negativnim. Takođe, kod pacijenata sa pozitivnim autoimunim antitelima su registrovani znatno viši nivoi interleukina-4 (IL-4), interleukina-10 (IL-10) i interleukina-6 (IL-6), dok su pacijenti sa negativnim antitelima imali značajno više vrednosti interferona-y (IFN-y) (P&lt; 0,05).

**Zaključak:**

Kod pacijenata sa hroničnim hepatitisom B postoje autoimuna antitela koja utiču na pokazatelje fibroze jetre i povezane citokine. Detekcija autoimunih antitela kod ovih pacijenata može da posluži kao koristan pokazatelj u proceni težine bolesti i prognozi ishoda.

## Introduction

In clinical practice, chronic hepatitis B is rather prevalent [1]. Data indicate that there are approximately 93 million chronic hepatitis B virus (HBV) infections and 20 million chronic hepatitis B patients [2][3][4]. Liver fibrosis indicators and cytokines can reflect the severity and clinical outcomes of chronic hepatitis B, although the incidence and progression of chronic hepatitis B are significantly influenced by autoimmunity [5]. Research [6][7][8] indicates that HBV can trigger an immune response in the body, generating autoimmune antibodies that cause damage to the liver. Consequently, it is crucial to identify autoimmune antibodies in patients with chronic hepatitis B. However, research on the relationships between autoimmune antibody levels, liver fibrosis indicators, and cytokines in patients with chronic hepatitis B is currently somewhat limited in China [9]. To explore the pathological mechanism of chronic hepatitis B and provide guidance for clinical treatment, autoimmune antibodies should be detected in patients, and their relationships with liver fibrosis indicators and cytokines should be analysed [10].

Hepatitis B virus (HBV) is the cause of chronic hepatitis B (CHB), a chronic liver illness [11]. Chronic infection can cause liver fibrosis, which can lead to liver cirrhosis and possibly hepatocellular carcinoma (HCC). Hepatic fibrosis is the liver's response to persistent damage and is characterised by excessive extracellular matrix (ECM) deposition [12][13][14]. The progression of this process indicates deterioration of the disease. Th1/Th2 cytokines play a significant role in immune responses and are crucial in the immunopathological mechanisms of chronic liver diseases. Th1 cytokines, such as interferon-γ (IFN-γ) and tumour necrosis factor-α (TNF-α), primarily promote cell-mediated immune responses and inhibit viral replication. Interleukin-4 (IL-4) and interleukin-10 (IL-10) are T2 cytokines that primarily participate in humoral immune responses by promoting B-cell differentiation and antibody production [15]. To explore the correlation between Th1/Th2 cytokines and hepatic fibrosis markers in CHB patients. In patients with CHB, Th1/Th2 cytokines may be contributing factors to liver fibrosis. A novel theoretical foundation and possible therapeutic targets for the treatment of CHB may be provided by an understanding of the role these cytokines play in liver fibrosis. Studying the relationships between Th1/Th2 cytokines and liver fibrosis indicators aids in the early identification and evaluation of liver fibrosis levels in CHB patients, guiding the selection of clinical treatment approaches [16]. If certain cytokines are closely related to liver fibrosis, they may serve as new biomarkers to assist existing diagnostic and monitoring methods, thereby increasing the accuracy and effectiveness of disease management [17][18][19][20].

This study detected the levels of major Th1/Th2 cytokines in the serum of CHB patients and conducted a correlation analysis in combination with liver fibrosis indicators (such as hyaluronic acid, laminin and type III procollagen peptide), aiming to reveal potential connections and provide concepts for early detection, evaluation of prognosis, and treatment of liver fibrosis in individuals with persistent heart failure on an individual basis.

## Materials and methods

### General information

Between March 2022 and March 2025, 102 patients with chronic hepatitis B were admitted, including 57 males and 45 females, who were chosen to serve as the observation group. The average age was 49.24±6.81 years, and the ages ranged from 18 to 71 years. Twenty-eight cases were mild, 53 cases were moderate, and 21 cases were severe. Extrahepatic manifestations included fatigue in 12 patients, arthralgia in 14 patients, jaundice in 8 patients, pruritus in 15 patients, and glomerulonephritis in 6 patients. A control group of 102 healthy adults, comprising 53 men and 49 women between the ages of 19 and 72, with an average age of 48.97±6.85 years, were chosen after undergoing physical tests over the same time period. The inclusion criteria for patients were as follows: aged 18 years; had a disease history of at least six months; and fulfilled the diagnostic criteria for chronic hepatitis B. No antibiotics, interferons or hormone drugs were taken. Be informed of and agree to this research. The exclusion criteria were as follows: liver cancer and liver cirrhosis; pregnant or lactating; blood diseases; acute or chronic liver injury caused by various factors; and autoimmune illnesses.

This study was approved by the Human Medical Research Ethics Committee (ZJJC18020022).

### Specimen collection

Each participant had six millilitres of fasting elbow venous blood extracted. The serum was isolated and kept for later use in a refrigerator at -20°C after being centrifuged for 15 minutes at 3,000 r-min^-1^ with an 8 cm centrifugation radius.

### Autoimmune antibody detection

Indirect immunofluorescence was used to identify serum antinuclear antibodies (ANAs), anti-mitochondrial antibodies (AMAs), and anti-smooth muscle antibodies (ASMAs). Serum samples were collected, redissolved at 37°C, and then diluted 1:100 with PBS. The experimental substrates included the liver tissues of monkeys, the stomach and renal tissues of Wistar rats, and HEP-2 cells. Bioslice technology was used to merge them into a reaction zone. An antibody tagged with fluorescein isothiocyanate was added following a 30-minute incubation period at 24°C to 30°C. A Japanese Olympus BX-51 fluorescent microscope was used to see the results after the plates had been cleaned and sealed. A positive outcome was indicated by the green fluorescence released by the cells or tissues. Western blotting was used to identify the anti-liver and kidney microsomal antibodies type I (LKM-1) and anti-liver-specific cytoplasmic antigen type I (LC-1). PBS was used to dilute the serum 1:100, and the matching antigen-coated blotting membrane strips were applied. For half an hour, the samples were agitated at room temperature on a shaker. Chromogenic reagent was applied to the membrane following washing. A standard schematic showing the positive band position was used to determine the membrane strip's positive result ten minutes later. German (Oumeng) Medical Laboratory Diagnostics GMBH supplied all of the reagent kits used.

### Laboratory biochemical testing methods

In all the research subjects, 10 mL of fasting venous blood was collected in the early morning and aliquoted into two types of pretreatment tubes: (1) EDTA-K anticoagulant tubes (BD Vacutainer®, catalogue number: 367525) were used for the detection of Th1/Th2 cytokines. After collection, the mixture was gently inverted and mixed 8 times, incubated at room temperature for 30 minutes, and centrifuged at 3000 rpm for 15 minutes at 4°C. The plasma was separated, aliquoted into sterile EP tubes, and frozen in a -80°C ultralow-temperature refrigerator (Thermo Scientific) for testing. (2) A common coagulation induction tube (BD Vacutainer®, catalogue number: 367812) was used for serological index detection of liver fibrosis. After standing at room temperature for 30 minutes to allow coagulation, the samples were centrifuged at 3000 rpm for 10 minutes to separate the serum, aliquoted and stored at -20°C. Cytokine detection was performed via a flow cytometry microsphere array (CBA) (BD Biosciences) with a human Th1/Th2 cytokine kit (BD CBA Flex Set, catalogue number: 558266). Quantitative concentrations of IFN-γ, IL-2, and TNF-α (Th1 type) and IL-4, IL-6, and IL-10 (Th2 type) in plasma (pg/mL) were measured. The experiment was carried out in strict accordance with the instructions, and data were collected on a BD FACSVerse flow cytometer. A standard curve was generated, and the concentration was calculated via FCAP Array v3.0 software. Four indicators of liver fibrosis (HA, LN, IV-C, PIII NP) were analysed via a chemiluminescence immunoassay (CLIA): a fully automatic chemiluminescence immunoassay analyser (Abbott ARCHITECT i2000SR) and matching original kits (HA: catalogue number: 8L77-25; LN: 8L78-25; IV-C: 8L79-25; (PIII NP: 8L80-25). The concentration of the indices in the serum (ng/mL) was determined via the double-antibody sandwich method. Each batch of tests included high- and low-value quality control products (Abbott quality control package, item number: 7K67-10).

### Liver function index detection

Serum liver fibrosis-related indicators of patients in the observation group. The Beijing Northern Institute of Biotechnology provided the reagent kits used, and the instructions for the reagent kits and instruments were strictly followed to perform the specific activities.

The liver function indicators detected in this study included ALT, AST, ALP GGT, total bilirubin (TBIL), direct bilirubin (DBIL), total protein (TP), albumin (ALB), and total bile acid (TBA). All commercial reagent kits from Nanjing Jiancheng Bioengineering Institute (Nanjing, China) were used. The specific item numbers and methodologies are as follows: ALT C009-2-1 (rate method), AST C010-2-1 (rate method), GGT C017-2-1 (γ-glutamyl p-nitroaniline method), ALP A059-2-1 (p-nitrophenol phosphate PNPP method), TBIL C019-1-1 (diazo method) (containing accelerator/caffeine reagent), DBIL C020-1-1 (direct diazo method), TP A045-2-1 (biuret method), ALB A028-2-1 (bromocrephenol green method), and TBA E003-1-1 (circulating enzyme method).

### Cytokine detection

The cytokines interleukin-4 (IL-4), interleukin-6 (IL-6), interleukin-10 (IL-10), and interferon-γ (IFN-γ) in the patients of the observation group were detected using a sandwich enzyme-linked immunosorbent test with two antibodies. Cytokine detection was performed via the flow microsphere array method. The CBA Human Th1/Th2 Cytokine Kit II (item number 551809; SAN Jose, California, USA) from BD Biosciences was used. It is used for the quantitative detection of IL-4, IL-6, IL-10 and IFN-γ (the kit also contains IL-2 and TNF indicators, which were not analysed).

The components in the kit (sample diluent, assay diluent, 10x wash buffer, recombinant human cytokine standard, capture microsphere mixture, and PE-labelled detection antibody) were all provided by BD Biosciences (Becton, Dickinson and Company, BD Biosciences, San Jose, CA, USA). BD FACSFlow Sheath Fluid (No. 342003): BD Biosciences (Becton, Dickinson and Company, BD Biosciences, San Jose, CA, USA). Compensation microbeads (BD CompBeads, item No. 552843; BD CompBeads Set Anti-Mouse Ig, к: BD Biosciences (Becton, Dickinson and Company, BD Biosciences, San Jose, CA, USA)) were used.

### Statistical analysis

Correlations between Th1/Th2 cytokine levels and liver fibrosis indicators were assessed via either Spearman rank correlation analysis or Pearson correlation analysis. To further confirm the reliability of the pertinent findings, we further performed multiple linear regression analyses to account for any confounding variables and investigate the separate impact of each cytokine on the severity of liver fibrosis. SPSS software will be used for all statistical analyses, along with two-sided tests.

## Results

### Comparison of the detection of autoimmune antibodies

The percentages of ANA-, ASMA-, LKM-1-, LC-1-, and AMA-positive samples in the observation group were 23.53%, 4.90%, 5.88%, 3.92% and 7.84%, respectively. Twenty-seven of them had at least one autoimmune antibody found, and the total positive rate was 27.45%. As shown in Table 1, the control group had a total positive rate of autoimmune antibodies of 3.92%, and the difference between the two groups was statistically significant (P<0.05).

**Table 1:** Comparison of positive rates of autoimmune antibodies.

Group category	n	ANA	ASMA	LKM-1	LC-1	AMA	Total positive
Observation group	102	24 (23.53)	5 (4.90)	6 (5.88)	4 (3.92)	8 (7.84)	28(27.45)
Control group	102	4 (3.92)	0 (0.00)	0 (0.00)	0 (0.00)	0 (0.00)	4(3.92)
x^2^ value	-	-	-	-	-	-	21.349
P value	-	-	-	-	-	-	0.001

### Comparison of liver function

Patients with positive autoimmune antibodies had much higher levels of AST and ALT than those with negative antibodies. In contrast, patients who were negative for antibodies had significantly higher levels of TP and ALB. Table 2 indicates that the differences were statistically significant (P<0.05).

**Table 2:** Liver Function Indicators between patients with positive and negative Autoimmune Antibodies (x̄ ± s).

Group category	n	AST/U ·L^-1^	ALT/U ·L^-1^	TP/g ·L^-1^	ALB/g ·L^-1^
Positive group	28	175.42±21.57	187.65±24.89	23.67±4.97	21.36±4.24
Negative group	74	134.31±17.22	149.48±19.36	31.84±5.26	29.75±5.02
t value	-	10.018	8.193	7.104	7.842
P value	-	0.001	0.001	0.001	0.001

### Comparison of liver fibrosis indicators

Patients with positive autoimmune antibodies in the observation group had significantly higher levels of HA, LN, PCIII, and IV-C than those with negative antibodies, and the difference was statistically significant (P<0.05), as shown in Table 3.

**Table 3:** Comparison of liver fibrosis index levels between patients with positive and negative autoimmune antibodies (x̄ ± s).

Group category	n	HA/mg ·L^-1^	LN/μg ·mL^-1^	PC/μg ·L^-1^	IV-C/μg ·L^-1^
Positive group	28	121.56±13.35	137.91±16.98	128.35±12.86	78.96±8.21
Negative group	74	98.37±11.48	115.42±12.73	109.53±12.17	67.35±7.99
t value	-	8.700	7.238	6.863	6.500

### Cytokine comparison

Patients with positive autoimmune antibodies in the observation group had significantly higher levels of IL-4, IL-6, and IL-10 than those with negative antibodies, while patients with negative antibodies had far higher levels of IFN-γ. The differences were statistically significant (P<0.05), as shown in Table 4.

**Table 4:** Comparison of cytokine levels between patients with positive and negative autoimmune antibodies (x̄ ± s).

Group category	n	IL-4/μg ·L^-1^	IL-6/μg ·L^-1^	IL-10/μg ·L^-1^	IFN- g/pg ·mL^-1^
Positive group	28	59.41±6.42	97.84±10.78	132.67±16.94	36.79±5.11
Negative group	74	45.38±5.76	78.63±8.89	115.42±13.15	49.62±6.38
t value	-	10.636	9.174	5.447	9.537
P value	-	0.001	0.001	0.001	0.001

## Discussion

Chronic hepatitis B patients may exhibit a range of symptoms and indicators [6][21]. Some patients may get liver cirrhosis, liver failure, or even primary liver cancer as the condition worsens [22]. Numerous factors contribute to the clinical development of chronic hepatitis B. Still, two main ones are host factors (age, sex, immunological state, etc.) and virological factors (HBV genotype, HBV expressed protein, HBV DNA load level, etc.) [23]. Instead of attacking liver cells directly, HBV triggers an immunological response in the body, which damages liver cells and induces inflammatory changes in liver tissue [24]. According to reports, the body's immune reaction to HBV is intimately linked to liver tissue deterioration in patients with chronic hepatitis B. Autoimmune antibodies are a form of immunoglobulin that attacks healthy human organs, tissues, and cells. They can harm the corresponding organs or target cells [25]. Other studies [26][27][28] have reported that hepatitis virus-infected individuals usually present with autoimmune manifestations [29]. Extrahepatic signs caused by an imbalance in immune tolerance mechanisms are also relatively common. Some patients have autoimmune antibodies in their bodies, which may be risk factors for organ dysfunction, histopathological damage, and aggravation of the condition [30].

Some scholars [31][32][33] believe that conducting autoimmune antibody testing on HBV-infected individuals can provide important references for clinical diagnosis, treatment plan formulation, and prognosis assessment. This study revealed that the positive rates of ANA, ASMA, LKM-1, LC-1 and AMA in the observation group were 23.53%, 4.90%, 5.88%, 3.92% and 7.84%, respectively, and the total positive rate was 26.47%. Moreover, in the observation group, patients with positive autoimmune antibodies had worse liver function indicators than did the control group, further confirming that autoimmune antibodies are present in the bodies of patients with chronic hepatitis B and that the degree of liver function impairment is substantially correlated with their expression.

Due to the influence of various factors, astrocytes in the liver are activated and proliferate, leading to liver fibrosis and aberrant proliferation of the liver's connective tissue [34]. An essential pathophysiological mechanism for the development of chronic hepatitis B into liver cirrhosis is hepatic fibrosis, which can result in aberrant alterations in liver structure and function. Serum levels of the liver matrix metabolites HA, LN, PCIII, and IV-C can indicate the progression of hepatic fibrosis. The patient's immune response is primarily responsible for the development and clinical outcomes of chronic hepatitis B. Helper T (Th) cells form the foundation of the immune system, and their function is primarily expressed through cytokine levels [35]. IFN-γ is a cytokine secreted by Th1 cells that can mediate immune responses related to local inflammation [36]. Cytokines are closely related to liver cell damage and liver fibrosis, significantly impacting the evaluation of liver fibrosis levels and the tracking of chronic hepatitis B [37].

In conclusion, patients with chronic hepatitis B have autoimmune antibodies that are associated with cytokines and markers of liver fibrosis to some degree. The detection of autoimmune antibodies has significant therapeutic implications for patients with chronic hepatitis B.

## Dodatak

### Funding

This work was supported by the Guiding Project of Quzhou Science & Technology Bureau (2024ZD006) and the Shen Xian Expert Workstation Fund (xk 2022-14).

### Acknowledgments

We thank all patients for participating in this study. Additionally, we thank Shandong University Qilu Hospital for providing the data.

### Conflict of interest statement

All the authors declare that they have no conflict of interest in this work.
